# Lisdexamfetamine-Induced Psychosis in a Patient With a Neurodevelopmental Disorder

**DOI:** 10.7759/cureus.53349

**Published:** 2024-01-31

**Authors:** Raghu Gandhi, Aimee Murray

**Affiliations:** 1 Department of Psychiatry, Abbott Northwestern Hospital, Minneapolis, USA; 2 Department of Psychiatry, University of Minnesota School of Medicine, Minneapolis, USA

**Keywords:** neurodevelopmental disorders, adhd, autism spectrum disorder, psychosis, stimulants

## Abstract

Individuals diagnosed with autism spectrum disorder (ASD) often experience a higher occurrence of comorbid attention deficit hyperactivity disorder (ADHD). Stimulant medications are frequently prescribed to manage ADHD. In rare instances, the use of stimulant medications has been linked to the development of psychotic symptoms. This is a case of a 13-year-old male diagnosed with ASD and comorbid ADHD, anxiety, and depression, who presented with an abrupt onset of psychosis, which manifested about a week after the initiation of lisdexamfetamine. The psychotic symptoms subsided upon discontinuation of lisdexamfetamine; however, there was a re-emergence of severe ADHD symptoms that proved resistant to non-stimulant medications. The patient experienced significant improvement without any recurrence of psychosis after being prescribed extended-release methylphenidate. Notably, there are no established clinical guidelines to assist in selecting one stimulant over another in the treatment of ADHD comorbid with ASD. The authors recommend considering the methylphenidate class of stimulants as a first-line treatment for ADHD in individuals with ASD, citing better tolerability compared to amphetamines.

## Introduction

Individuals with autism spectrum disorder (ASD) have a higher prevalence of common mental health disorders than typically developed individuals [1]. Patients with ASD are sensitive to psychotropic medications, and they are at increased risk for psychosis compared to the general population at baseline [2]. The pooled estimate of the lifetime prevalence of attention deficit hyperactivity disorder (ADHD) in ASD is as high as 40.2% [3], with stimulants being prescribed as a first line of treatment for these symptoms. In rare cases, stimulants are associated with psychosis, and amphetamines are associated with a higher risk (0.2%) compared to methylphenidate (0.10%) [4]. At this time, no guidelines exist for recommending one particular class of stimulant over another. We present a case of a 13-year-old adolescent male who had improvement in psychotic symptoms following lisdexamfetamine to methylphenidate stimulant switch.

## Case presentation

A 13-year-old male, with a psychiatric history of ASD, ADHD, anxiety, and depression, was presented to outpatient child psychiatry services for new-onset psychotic symptoms and increased verbal and behavioral aggression. The symptoms began three weeks before this clinic visit. Psychotic symptoms included visual and auditory hallucinations, paranoid delusions, and bizarre behaviors. His parents reported that he was living in “virtual reality,” where he “acted like a cat.” He was making hissing noises, trying to claw at people's faces. He was seeing cartoons coming out of the ceiling telling him to do things, and he would mumble to himself for extended periods. He had "zombie vision," which made things "twisted" and people's eyes big. He had a poor sleep at night as he was worried about recurrent bad dreams about "Mooney owls" and the “Mooney owl queen" coming out of the dark screen of the television or dark window. He stated he had made peace with Mooney Owls after agreeing “not to try to steal their eggs,” but the queen Mooney Owl might still bother him with "voodoo-biting bats" that would attack his head and fingers. At school, he acted paranoid. He complained about his peers poking at his back, stomach, and butt. He heard their voices telling him, "You don't belong here.” His behavior was unpredictable. He slapped the school principal. He had difficulty transitioning from one task to another. In the clinic, mental status examination was significant for increased psychomotor activity, pressured speech, paranoid delusions, visual hallucinations, and thought perseveration on “Mooney owls.” Insight and judgment were limited. The patient has been prescribed multiple medications, which included lisdexamfetamine 40 mg qAM, guanfacine 1 mg TID, aripiprazole 5 mg qAM, fluoxetine 15 mg qAM, clonidine 0.1 mg qHS, and trazodone 100 mg qHS. He had some significant medication adjustments recently, and four weeks before this visit, methylphenidate extended-release 36 mg was switched to lisdexamfetamine 40 mg for attentional difficulties and impulsivity. Then, two weeks after that risperidone 2 mg was switched to aripiprazole 5 mg for these psychotic symptoms. The patient was on methylphenidate extended-release 36 mg for three months and risperidone for the past two months. A thorough first episode psychosis workup, including complete blood count, comprehensive metabolic panel, serum B12, urine toxicology screen, thyroid stimulating hormone, HIV test, ESR, CRP, antinuclear antibody test, and free urine cortisol, and an MRI brain study yielded unremarkable results. The patient had been diagnosed with ASD at age three and ADHD at age eight, with symptoms of ASD involving deficits in social communication and repetitive restricted behaviors and interests. Anxiety about bee bites and the well-being of family members, coupled with occasional feelings of sadness and hopelessness, were reported. No significant medical and substance use history.

During this clinic visit, lisdexamfetamine dose was decreased from 40 mg to 30 mg with a close follow-up in two weeks. The patient had improvement in psychotic symptoms such as hallucinations and paranoia, but perseveration about Mooney Owls persisted. Further reduction in lisdexamphetamine dose from 30 to 10 mg led to rebound ADHD symptoms such as significant impulsivity, poor emotional regulation with frequent meltdowns, and distractibility. At that point, lisdexamphetamine 10 mg was switched to methylphenidate extended-release 10 mg. This medication change led to the remission of psychotic symptoms in two weeks. The patient’s affect was bright during subsequent follow-ups, with no restlessness, pressured speech, and perseverative thinking. At the time of the last follow-up, the patient was on methylphenidate extended-release 10 mg q AM, guanfacine 1 mg TID, aripiprazole 5 mg qAM, fluoxetine 15 mg qAM, clonidine 0.1 mg qHS, and trazodone 100 mg qHS.

## Discussion

The differential diagnosis is complicated by the comorbid presentation of ASD. Autism and schizophrenia share genetic and environmental vulnerabilities [5,6]. The symptom profile can overlap, and it can be challenging to determine what underlying pathology contributes to social-emotional concerns, executive functioning deficits, and disordered speech and behavior [5-7]. In this case, the patient’s difficulties reading social cues and communication challenges related primarily to autism might explain what appears to be delusional thoughts and paranoia. Idiosyncratic and perseverative thinking and expressive difficulties found in some people with autism might warrant the peculiar descriptions of the “Mooney Owls” and the reports of living in a virtual world. It is also possible that someone with autism might present with acute transient psychotic-like symptoms due to severe stress [8].

 A critical difference between autism and a primary psychotic disorder is perceptual abnormalities or beliefs distinct from baseline or functional declines from baseline [5]. Caregivers can often reliably identify these differences [5]. Autism was ruled out as an explanation for the presenting symptoms. In this case, the patient’s parents described a change from baseline. In addition, the authors ruled out the stress hypothesis because he continued to describe the experiences even after he no longer had contact with the peers he described as bullying him.

Schizophrenia was considered. The rates of schizophrenia in populations with autism are higher than the general population, 4% versus 1%, respectively [9]. His symptoms presented earlier than typical for schizophrenia, but people with childhood-onset schizophrenia have a 25-28% chance of having ASD [5]. Schizophrenia was unlikely because his symptoms had what appeared to be an acute onset, and there is extensive evidence that the course of schizophrenia unfolds in stages [10]. Further, his first episode of medical workup was negative.

Medication-induced psychotic disorder was also considered. The presentation of the symptoms was acute, and there were no new-onset negative symptoms, which is more consistent with medication-induced psychosis [11]. This was punctuated with a significant reduction in psychotic symptoms when lisdexamphetamine was reduced from 40 mg to 30 mg, followed by switching to a different stimulant. The quick response to a decrease in lisdexamphetamine is also consistent with medication-induced psychotic disorder [12].

Research investigating the comorbidity of ADHD and ASD found prevalence rates ranging between 53% and 78% [12]. Stimulants remain the first-line treatment for ADHD and, in rare cases, are associated with psychotic symptoms [13]. Psychotic symptoms range from paranoid delusions to confusion, increased aggression, and visual, auditory, tactile, and somatic hallucinations [14]. According to a recent study by Moran et al., the rate of psychosis after starting stimulant medication for ADHD is low, one in 660 adolescents and young adults. The rate is twice as high when using amphetamines compared to methylphenidate (0.10% among patients receiving methylphenidate versus 0.21% among patients receiving amphetamines) [4]. Current guidelines by the American Academy of Pediatrics (AAP) indicate equal efficacy for methylphenidate and amphetamines. Although methylphenidate is the most frequently prescribed stimulant in many countries, data from private insurance claims show that amphetamines are more commonly prescribed in the United States [15]. Both classes of stimulants, methylphenidate, and amphetamine, enhance the release of dopamine from neurons and inhibit dopamine reuptake into presynaptic terminals [16]. However, dopamine release is four times higher with amphetamine compared to methylphenidate [17]. Cortese et al. conducted a meta-analysis to compare the efficacy and tolerability of ADHD medication in children, adolescents, and adults. Although amphetamines were found to be the most efficacious in all age groups, they were less tolerated in children [15]. The finding of an increased incidence of psychosis in patients taking amphetamines compared with methylphenidate suggests that methylphenidate should be considered the first-line treatment for ADHD, particularly in children and young adolescents.

## Conclusions

The incidence of psychosis is higher in people with ASD than in the general population. Fifty percent of people with ASD have ADHD symptoms requiring medication management. The risk of psychosis is slightly higher in individuals taking amphetamines than methylphenidate. There are no guidelines about considering a particular class of stimulants over others when treating ADHD symptoms in patients with ASD. Authors recommend that stimulants belonging to the methylphenidate class should be considered over amphetamines for treating ADHD comorbid with ASD. Careful differentiation is critical in these cases, and clear timelines and collateral information may be critical to establishing the differential diagnosis. Further research should be conducted to investigate the risk of psychosis associated with the use of stimulants in patients with autism spectrum disorder.
